# EphrinA5 Signaling Is Required for the Distinctive Targeting of Raphe Serotonin Neurons in the Forebrain

**DOI:** 10.1523/ENEURO.0327-16.2017

**Published:** 2017-02-06

**Authors:** Teng Teng, Afsaneh Gaillard, Aude Muzerelle, Patricia Gaspar

**Affiliations:** 1 Inserm UMR-S 839, 75005, Paris, France; 2 Université Pierre et Marie Curie, Paris, France; 3 Institut du Fer à Moulin, Paris, France; 4 Inserm 1084, Poitiers, France; 5 Université de Poitiers, Poitiers, France

**Keywords:** amygdala, culture, Ephrin, hypothalamus, in utero electroporation, olfactory bulb

## Abstract

Serotonin (5-HT) neurotransmission in the brain relies on a widespread axon terminal network originating from the hindbrain raphe nuclei. These projections are topographically organized such that the dorsal (DR), and median raphe (MnR) nuclei have different brain targets. However, the guidance molecules involved in this selective targeting in development are unknown. Here, we show the implication of ephrinA5 signaling in this process. We find that the *EphA5* gene is selectively expressed in a subset of 5-HT neurons during embryonic and postnatal development. Highest coexpression of *EphA5* and the 5-HT marker Tph2 is found in the DR, with lower coexpression in the MnR, and hardly any colocalization of the caudal raphe in the medulla. Accordingly, ephrinA induced a dose-dependent collapse response of 5-HT growth cones cultured from rostral but not caudal raphe. Ectopic expression of ephrinA3, after *in utero* electroporation in the amygdala and piriform cortex, repelled 5-HT raphe fiber ingrowth. Conversely, misplaced DR 5-HT axons were found in ephrin A5 knockout mice in brain regions that are normally only targeted by MnR 5-HT axons. This causes an overall increase in the density of 5-HT innervation in the ventromedial hypothalamus, the suprachiasmatic nucleus, and the olfactory bulb. All these brain areas have high expression of ephrinAs at the time of 5-HT fiber ingrowth. Present results show for the first time the role of a guidance molecule for the region-specific targeting of raphe neurons. This has important implications to understand how functional parsing of central 5-HT neurons is established during development.

## Visual Abstract

**Figure:**
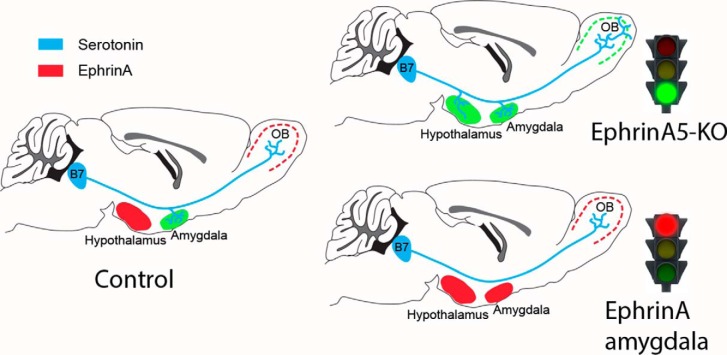

## Significance Statement

This study shows for the first time the role of a guidance molecule for region-specific serotoninergic innervation. We establish that Eph-ephrinA signaling acts as a repulsive signal to target 5-HT axons originating from different raphe nuclei. EphA5 expression varies across raphe nuclei, correlating with different repulsive actions of ephrinA on 5-HT axons. EphrinA overexpression inhibits 5-HT innervation of the amygdala while EphrinA5 loss of function causes a mis-targeting of dorsal raphe 5-HT axons in the olfactory bulb and hypothalamus. This shows a new role of ephrinA signaling in 5-HT target selection and is likely to have important functional consequences.

## Introduction

Serotonin (5-hydroxytryptamine, 5-HT) neurotransmission is implicated in a large number of physiological functions, raising the question of a division of labor among the different hindbrain nuclei that synthesize 5-HT (Calizo et al., 2011; Hale and Lowry, 2011; Kiyasova and Gaspar, 2011). 5-HT–synthesizing neurons are distributed into several raphe nuclei in the hindbrain that have been parsed according to anatomical and physiological criteria. Individual raphe nuclei target different brain regions and, consequently, are involved in different functions. For instance, the caudal 5-HT raphe nuclei (B1–B3 cell groups), which are located in the medulla and project to the brainstem and spinal cord, are implicated in motor control and neurovegetative functions (Schmidt and Jordan, 2000; Pflieger et al., 2002; Brust et al., 2014). Conversely, 5-HT neurons of the rostral raphe, which are located in the pontine region of the hindbrain (B5–B9 cell groups) and project to the forebrain, have been involved in diverse higher brain functions, such as mood, learning, and social behaviors such as aggression and maternal behavior (Deakin and Graeff, 1991; Lucki, 1998; Trowbridge et al. 2011). Within these broad raphe divisions, further anatomical and functional distinctions can be made; for instance, the rostral raphe cluster comprises neurons of the dorsal raphe (DR = B6 + B7) and the median raphe (MnR = B5 + B8) groups that are implicated in different brain functions (Jacobs and Azmitia, 1992; Teissier et al., 2015; Fernandez et al., 2016) and innervate complementary targets in the telencephalon (Vertes et al., 1999; Muzerelle et al., 2016). This organization suggests that specific axon guidance molecules could orient the 5-HT–containing axons to specific targets, although the underlying molecular mechanisms are largely unknown (Kiyasova and Gaspar, 2011).

Previous transcriptome profiling of developing 5-HT raphe neurons identified distinct expression profiles between the rostral pontine and the caudal medullary raphe cell groups (Wylie et al., 2010). Among these differentially expressed genes, a number of axon guidance molecules, such as Eph receptors and their ephrin ligands, are potential candidates for the selective guidance of 5-HT neuron subsets. The properties of EphA receptors as short-range guidance factors make them attractive candidates for selective axon targeting (O’Leary and Wilkinson, 1999; Klein and Kania, 2014). Eph-ephrin signaling has indeed been involved in many functions, one of the best known being its role in establishing topographic maps in several sensory systems (Prakash et al., 2000; Miko et al., 2007). Moreover, the EphA ligand, ephrinA5, was suggested to be involved in the development of the dopaminergic neurons, based on its dynamic developmental expression patterns (Deschamps et al., 2009, 2010; Cooper et al., 2009; Prestoz et al., 2012).

In the present study, we demonstrate a role for Eph-ephrinA signaling in developing 5-HT raphe neurons to repel axon growth of a subset of 5-HT neurons. We find that ephrinA5 is required for the exquisite differential targeting of the DR and MnR axons in the olfactory bulb, amygdala, and hypothalamus. These observations establish for the first time a role of Eph-ephrinA signaling to organize the broad topography of a monoaminergic system.

## Materials and Methods

### Animals

Most experiments (gene expression, cell cultures, electroporations) were performed on mice of the Swiss background (RjOrl:SWISS) purchased from a commercial breeder (Centre d’Elevage R. Janvier). Embryonic day 0 (E0) was defined as the plug date and postnatal day 0 (P0) as the date of birth.

The Pet1-Cre::RCE-GFP mouse line was used for reverse-transcription qualitative PCR (RT-qPCR) analyses. ePet1-Cre mice (Scott et al., 2005) in which the serotonergic specific promoter of the Pet1 gene that controls Cre expression was crossed to the RCE-GFP mouse line where enhanced green fluorescent protein (eGFP) is conditionally expressed under the Rosa-26 promoter (Sousa et al., 2009). Pet1-Cre::RCE-GFP mice were bred in our local facility, and brains were collected from P5, P15, and adult mice.

The ephrinA5 knockout (KO) mouse line (Frisén et al., 1998) was maintained on a C57Black6 background (Deschamps et al., 2009). Briefly, these mice have a PGK-neocassette replacing the 5′ acceptor splice site and the sequences encoding amino acid residues 42–129. The PCR primers for genotype are as follows: primer 1 (TCCAGCTGTGCAGTTCTCCAAAACA) and primer 2 (ATTCCAGAGGGGTGACTACCACATT) for wild-type sequences (397 bp) and primers 1 and 3 (AGCCCAGAAAGCGAAGGAGCAAAGC) for mutant sequences (513 bp).

All experiments were performed in compliance with the standard ethics guidelines (European Community Guidelines and French Agriculture and Forestry Ministry Guidelines for Handling Animals, decree 87849). All efforts were made to reduce the number of animals used and their suffering. Male and female mice were used indiscriminantly in all experiments.

### Histology

#### Section preparation

Brains of Swiss mice were collected at E12, E14, E16, E17, E18, P0, P5, P10, and P15 and from adults aged >8 weeks.

Mice P5 or younger were anesthetized on ice. Mice older than P5 were anesthetized with pentobarbital, 25 mg/kg, and xylazine, 5 mg/kg. Fixation was either with immersion (E14, E16) or perfusion (>E16) with 4% paraformaldehyde (PFA; 4% PFA in 0.12 m phosphate buffer, pH 7.4). Dissected brains were postfixed for 2 h (embryonic ages) or overnight (all postnatal ages) before cryoprotection in 10% sucrose and freezing in isopentane cooled with dry ice at –45° to –55°C. In some *in situ* hybridization (ISH) protocols, no postfixation was performed. Frozen brains were then cut with a cryostat to either coronal or sagittal 20-μm-thick sections and collected as series of six. Frozen sections were stored at –80°C before immunohistochemistry or ISH. Some brains were processed as floating sections and sectioned with a freezing microtome. In this case, the brains were cryoprotected in 30% sucrose and frozen directly on the platform of the cryotome at –40°C; serial 40-μm-thick coronal sections were collected in 1× PBS with 0.01% sodium azide. Sections were stored at 4°C before processing.

#### ISH

ISH was used to analyze EphA and ephrinA expression. Digoxigenin-labeled mRNA probes were transcribed from mouse EphA3, EphA4, EphA5, EphA7, Efna2, Efna3, and Efna5 cDNAs. Sense and antisense digest enzymes and polymerases of these probes are listed in Table 1. Sections were air-dried for at least 2 h under a hood. Specific antisense RNA probes (0.1–1 μg/ml) were mixed with hybridization buffer (50% formamide, 10% dextran sulfate, 1× Denhardt’s, 5× SSC, and 250 μg/ml transfer RNA) and incubated at 52°C, 58°C, or 65°C for 10 min. Mixed hybridization buffer (350 µl) was added to each section, covered with a coverslip, and incubated overnight at the same temperature. The sections were washed with PBS and PBS Triton 0.1% and incubated with anti-digoxigenin (anti-Dig; 1/1000) at 4°C overnight. Sections were washed with 1× PBS and NTMT (Tween 10%; Tris-HCl, pH 9.5, 1 m; MgCl_2_, 1 m; NaCl, 5 m; H_2_O) buffer and incubated at 37°C with NBT+BCIP or fast red (TR/naphthol AS-MX Tablets, Sigma-Aldrich, F4523-50 SET) to reveal the reaction. Duration of the revelation (2–24 h) was determined empirically according to the sensitivity of the probes and the concentration of the anti-Dig solution. The signal was checked with bright-field or fluorescence microscopy. Duration was kept identical for a given experiment (e.g., time course of expression). The sections were washed with 1× PBS and mounted in mowiol-Dabco (25 mg/ml) or processed with immunohistochemistry.

**Table 1.** List of mRNA probes

	Restriction enzyme		Polymerase	
Probe	Sense	Antisense	Sense	Antisense
EphA3	HindIII	EcoRI	T7	T3
EphA4	SacI/SacII	XhoI/BamHI	T7	T3
EphA5	Xbal	BamHI	T3	T7
EphA7	BamHI	XhoI	T7	SP6
Efna2	HindIII	EcoRV	SP6	T7
Efna3	XhoI	BamhI	T7	SP6
Efna5	HindIII	XbaI	T3	T7

#### Immunohistochemistry

Immunohistochemistry was performed either on alternate series of sections or in combination with ISH. Sections were washed in PBS, then in PGT (PBS with 0.2% gelatin and 0.25% Triton X-100) four times for 15 min. Sections were incubated overnight at 4°C with the following primary antibodies: anti-Tph2 (mouse monoclonal, 1/1000, Sigma-Aldrich), anti-5-HT (rabbit polyclonal, 1/1000, Sigma-Aldrich), anti–serotonin transporter (anti-SERT; rabbit polyclonal, 1/1000, Calbiochem). For fluorescence microscopy, sections were then incubated for 2 h at room temperature with the following secondary antibodies: donkey anti-rabbit 488 (1/200, The Jackson Laboratory), donkey anti-rabbit Cy3 (1/200, The Jackson Laboratory), donkey anti-mouse 488 (1/200, The Jackson Laboratory), donkey anti-mouse Cy3 (1/200, The Jackson Laboratory), or phalloidin 594 (1/40, Invitrogen). Sections were rinsed in PB, mounted in mowiol-Dabco (25 mg/ml), and stored at 4°C.

#### RT-qPCR

Brains of Pet1-Cre::RCE-GFP mice were collected at P5, P15, and adult (6 weeks). Brains were kept in 1× PBS on ice and sectioned in the coronal plane with a tissue chopper to 300-μm-thick sections. The DR was microdissected under a fluorescent microscope (Zeiss-MV10) and collected into 5-ml tubes, which contained 2 ml of cold 1× PBS; a cortical hemisphere was collected as a positive control. To obtain enough tissue for RNA isolation from the raphe, four cases were pooled together for each sample analyzed. Tissue was either directly processed for RNA isolation or fast frozen at –80°C. RNA isolation from tissue was done with Trizol reagent (Sigma-Aldrich). Samples were weighed (≤50 mg) before homogenization, PBS was removed extensively, and 1 ml of Trizol was added. Tissue was homogenized, and homogenates were transferred to 2-ml Eppendorf tubes with 0.2 ml of chloroform, gently mixed for 15 s, and centrifuged at 12000 × *g*, for 15 min at 4°C. The upper phase was collected, and RNA was precipitated with 0.5 ml isopropyl alcohol, incubated at room temperature (5 min), and centrifuged at 12000 × *g* for 10 min at 4°C. After removal of the supernatant, the RNA pellet was air-dried and dissolved in 100 μl Milli-Q water and stored at –80°C.

Possible DNA contamination was cleared using DNase I (Thermo), and RT-PCR was done with the SuperScriptII kit (Invitrogen). qPCR was performed with the Thermo SYBR Green Mix kit according to the manufacturer’s instructions. Primers are listed in Table 2.

**Table 2.** List of RT-qPCR primers

Primer	mRNA Variant 1	Product length	Forward primer	Reverse primer
EphA3	NM_010140	116	TGCGGGACTGTAACAGCATT	CGTGAACTGATGCTCTCGGA
EphA4	NM_007936	90	GAGGCTCCTGTGTCAACAACT	AGTTGCCAATGGGTACCAGC
EphA5	NM_007937	98	TTGGCTGTTGACCAGTTGGA	GTCCTCCAGGAAGGCTGTTG
EphA6	NM_007938	90	ACTGAAATCCGTGAGGTGGG	GACTGAGACCAGAGCGATGC
EphA7	NM_010141	98	TCCTCCTTAGTCGAGGTCCG	GCCACTCTCCTTCTGCACTG
EphA8	NM_007939	95	CATTGCTTTCCGCACGTTCT	TCCAGTAGGGTCGTTCACCA

#### Raphe culture and collapse assay

E12 embryos were collected from Swiss timed-pregnant dams. Embryonic hindbrains were rapidly dissected as an open book in ice-cold 1× PBS. The rostral and caudal raphe were separated based on anatomical landmarks. The dissected raphe was further cut into 200-μm explants with a tissue chopper. Explants were placed onto polylysine/laminin-coated glass coverslips (Marienfield, 0111540) in four-well culture boxes (Nunclon, 176740) in DMEM F-12 medium to which BSA (1%), penicillin/streptomycin, glutamine (200 mm), and glucose (50%) were added. Explants were cultured for 3–4 d at 37°C, in 5% CO_2_. For the collapse assay, ephrinA5 (R&D System, 374-EA) was added at different concentrations (50, 250, 500 mm) for 1 h. Explants were then quickly washed in PBS, fixed in buffered 4% PFA for 30 min, and washed extensively before 5-HT immunocytochemistry (anti–5-HT rabbit polyclonal, 1/1000, Sigma-Aldrich) and phalloidin 594 (1/40, Invitrogen) staining.

#### Quantification of the collapse assay

Explants were imaged with a fluorescence microscope. Only round-shaped explants that contained 5-HT neurons were quantified. More than 100 growth cones from three explants for each condition were counted using a 63×/1.25 objective. The number of collapsed/noncollapsed growth cones was counted for both 5-HT and non–5-HT axons, comparing 5-HT immunostaining with phalloidin staining. Results from three independent experiments were used to calculate the mean ratio ± SEM per condition.

#### Anterograde tracing

We used an adeno-associated virus (AAV1.CAG.TdTomato.WPRE.SV40 ref: AV-1-PV3365, Penn Vector Core) to express TdTomato fluorescence in the DR neurons and projections. A single injection (20 nl of the virus undiluted) was done in the DR using glass-pulled capillaries (5-000-1001-X10, Drummond; puller model 720, Kopf, heat of 14.5 and solenoid 2). DR stereotaxic injections were performed as previously described (Muzerelle et al., 2016). Adult ephrinA5 KO mice and control littermate (+/+ or +/–) mice were anesthetized with ketamine (150 mg/kg)/xylazine (10 mg/kg). Animals were positioned on a foam board horizontally, the head was fixed and kept horizontal, and bregma coordinates were measured to calculate the position of the injection site and the angle of stereotaxic arm. To target the DR, the following coordinates were used: antero-posterior, 0.5 mm to lambda; medio-lateral, 1 mm; dorsoventral, –3.2 mm. The animals were kept for 3 weeks and were perfused by 4% PFA. Brains were processed as described above, collecting serial 50-μm coronal sections throughout the brain.

#### *In utero* electroporation of EphrinA construct

GFP-Efna3 was subcloned into the vector pCIG-TdTomato. The pCIG-TdTomato vector without the insert was used for the control condition. Both plasmids were purified by Qiagen EndoFree Plasmid Maxi kit, and stored at a final concentration of 2.5μg/μl.

To target gene expression in the amygdala, we followed a previously described *in utero* electroporation protocol (Remedios et al., 2004; Huang et al., 2014). The plasmids (1 μg/μl) were mixed with 1% Fast Green (F7252, Sigma-Aldrich) and injected into one of the lateral ventricles of E12.5 embryos with glass-pulled micropipettes. A 3-mm-diameter electrode (LF650P3, Bex Co.) was placed toward the caudal and ventral part of the telencephalon. Six electric pulses (30 V, 50-ms pulse length) with 950-ms intervals were applied using an electroporator (CUY21, Bex Co.). After delivery, foster mothers delivering at the same time as the experimental subjects adopted the newborn pups. The electroporated pups were perfused at P5 and processed for immunocytochemistry as described above.

#### Image acquisition

For bright-field microscopy, histological sections were imaged using a slide scanner (Nanozoomer 2.0-HT C9600, Hamamatsu) 20× objective or captured with a CoolSnap camera mounted on a bright-field microscope (Provis, Olympus). For illustration purposes, images from the nanozoomer were exported in Tiff format using the NDP View2 software (Hamamatsu).

For fluorescence microscopy, images were acquired with a Leica DM 6000B system using a 40×/0.70 oil objective (tissue cultures) or a Leica SP5 confocal system (colocalization and fiber density analyses) equipped with an Argon laser (for the 488-nm excitation), a diode 561 nm and HeNe 633 nm. *Z*-stacks of confocal images were acquired at 1024 × 1024-pixel resolution, with a pinhole set to one Airy unit and optimal settings for gain and offset.

#### Image analyses

EphA5-Tph2 colocalization was analyzed on P5 brains processed from three independent experiments. Cryostat sections through the brainstem (20 μm thick) were collected as series of six. One series was processed for combined EphA5 HIS (Fast Red Chromogen), Tph2 immunohistochemistry (revealed with Alexa 488), and DAPI and imaged with a confocal microscope. These three fluorochromes were sequentially acquired with a 40×/1.25-NA Plan-Apochromat objective at three different rostrocaudal levels through the raphe (Paxinos Atlas, levels: bregma –4.3, –4.6, and –4.9 mm). The whole hindbrain area containing Tph2^+^ neurons was acquired including the different subdivisions of the DR in the dorsal part (DRD), the ventral part (DRV), the lateral wings (DR-LW), and the caudal part (DR-C, B6). Acquisitions included the MnR, which comprises the B8 and B5 cell groups, and the supralemniscal B9 cell groups. Confocal stacks were analyzed with ImageJ. A 150 × 150-μm square mask was used for random selection of counting areas. Three random selections were positioned on each distinct 5-HT subnucleus. A cell counter plugin was used to count the Tph2^+^ cells on individual confocal sections, EphA5^+^ cells, and colocalized neurons. It should be noted that in contrast to BCIP, the Fast Red used as a chromogen for ISH mRNA revelation generally diffuses into the nucleus of labeled cells, in contrast to the Tph2 labeling that remains in the cytoplasm (e.g., Fig. 2 *C*, Fig. 3).

Fiber density was analyzed in two different ways, anterograde tracer injections and *in utero* electroporation. In the olfactory bulb and amygdala, confocal images were acquired at 4-μm intervals over 20 μm in *z*. A maximum *z*-projection of the image stacks was performed with ImageJ. Then, a circular mask of 20-μm diameter was used for random sample selection. All labeled fibers that crossed the edge of the mask were counted with a cell counter to compute linear densities. In the hypothalamus, where there is a high density of 5-HT innervation, single confocal images were analyzed. Confocal 16-bit images were copied to ImageJ with an 8-bit format and processed as described (Kiyasova et al. 2011). Subtraction of the background was done with a 20-pixel rollerball, and a binary image was obtained after applying a fixed-range threshold for all the images. The nuclear region of interest (ROI) was delimited, and the area occupied by the labeled fibers was measured within a circular mask (100-μm diameter). The mask was randomly placed in three to five different locations over the structure, to calculate a mean density value per area and per animal.

### Statistical analyses

All the statistical analyses applied were performed with GraphPad Prism 6. One-way ANOVA was performed for the qPCR, colocalization, and collapse assays. To evaluate differences between any of two samples, Tukey’s multiple comparisons was performed. Student’s *t*-test was performed for intergroup comparisons in the fiber density analyses. Unpaired *t*-test was used for comparison of independent samples, and paired *t*-test was done for analyses comparing ipsilateral and contralateral innervation in the same case. Data are expressed as mean ± SEM; *p* < 0.05 was considered significant. Statistical tests are shown in Table 3.

**Table 3.** Statistical analysis

Line	Data structure	Type of test	Power
a, (Fig. 2A)	Homoscedastic	One-way ANOVA Tukey’s multiple comparisons test	0.7792
b1, (Fig. 3D)	Homoscedastic	One-way ANOVA Tukey’s multiple comparisons test	0.8335
b2, (Fig. 3E)	Homoscedastic	One-way ANOVA Tukey’s multiple comparisons test	0.7097
c1, (Fig. 4C)	Homoscedastic	One-way ANOVA Tukey’s multiple comparisons test	0.9714
c2, (Fig. 4D)	Homoscedastic	One-way ANOVA Tukey’s multiple comparisons test	0.9783
c3, (Fig. 4E)	Homoscedastic	One-way ANOVA Tukey’s multiple comparisons test	0.266
c4, (Fig. 4F)	Homoscedastic	One-way ANOVA Tukey’s multiple comparisons test	0.934
d1, (Fig. 5D)	Homoscedastic	Paired *t* test (two tailed)	1
d2, (Fig. 5E)	Homoscedastic	Paired *t* test (two tailed)	1
e1, (Fig. 6F)	Homoscedastic	Unpaired *t* test (two tailed)	1
e2, (Fig. 6G)	Homoscedastic	Unpaired *t* test (two tailed)	1
e3, (Fig. 6H)	Homoscedastic	Unpaired *t* test (two tailed)	0.052
f1, (Fig. 7D)	Homoscedastic	Unpaired *t* test (two tailed)	1
f2, (Fig. 7E)	Homoscedastic	Unpaired t test (two tailed)	1
g1, Fig. 7I	Homoscedastic	Unpaired *t* test (two tailed)	1
g2, Fig. 7J	Homoscedastic	Unpaired *t* test (two tailed)	1

Statistical calculations were done with GraphPad Prism 6. Powers of each experiment were calculated by an online power calculator: https://www.anzmtg.org/stats/PowerCalculator.

## Results

### Expression of EphA receptors in the raphe nuclei

In rodents, 5-HT neurons of the hindbrain start extending axons by E12, reach most of their forebrain targets between E15 and P1, and start arborizing in these targets over the following postnatal days (Lidov and Molliver, 1982, in rats; Kiyasova and Gaspar, 2011, in mice).

**Fig. 1.**
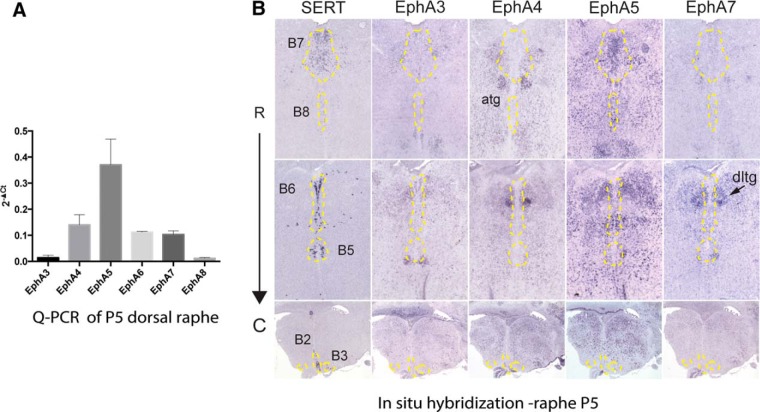
EphA receptor gene expression in the developing mouse raphe. ***A***, qPCR of EphA3, EphA4, EphA5, EphA6, EphA7, and EphA8 mRNAs in DR extracts from P5 mice (*n* = 4 mice/experiment). Relative mRNA expression was calculated as 2^–ΔCt^ (delta of cycle threshold). Data are presented as mean ± SEM from three independent experiments. ***B***, *In situ* hybridization of SERT, EphA3, EphA4, EphA5, and EphA7 mRNAs is shown at three different rostro-caudal levels of the raphe nuclei, including DR (B7), caudal DR (B6), rostral MnR (B8), caudal MnR (B5), raphe pallidus (B1), obscurus (B2), and magnus (B3). Coronal serial sections (20 μm thick) were labeled with the five different probes. Localization of 5-HT neurons as revealed by SERT expression was outlined with dashed yellow lines that were transferred to the consecutive sections on the series. This shows that only EphA5 labeling coincides clearly with the contours of B7 and B8. EphA4 and EphA7 are also strongly expressed in the brainstem, but signal is mainly detected in cell groups such as the anterior tegmental nucleus (atg), the dorsal tegmental nucleus (dtg), or the inferior olive (io) that come very close to the raphe. Scale bar = 250 μm.

We began by screening the expression of EphA receptors at P5, when the 5-HT axons are still actively growing and branching in their targets (Lidov and Molliver, 1982) and where the DR and the MnR can be clearly individualized. qPCR analyses were performed on microdissected DR from Pet1-GFP mice (Scott et al., 2005). *EphA3*, *A4*, *A5*, *A6*, *A7*, and *A8* mRNAs were analyzed, using GAPDH as a housekeeping gene. This analysis showed that *EphA5* is the most abundant EphA receptor in the DR at P5, with lower expression of *EphA4*, *EphA6*, and *EphA7* and no detectable expression of *EphA3* and *EphA8* (Fig. 1*A*). To determine the cellular localization of the *Eph* genes, ISH was performed on consecutive serial coronal sections through the raphe, using specific mRNA probes to EphAs (Table 1) and SERT used as a marker of the 5-HT raphe neurons. This showed that among the Eph genes examined, only *EphA5* was clearly localized in the DR and the MnR containing 5-HT neurons, but with no visible expression in the raphe magnus, obscurus, and pallidus (the B1–B3 raphe cell groups; Fig. 1*B*). The other EphA genes, *EphA4* and *EphA7*, were not detectable in any of the raphe nuclei but were localized to nuclei, such as the dorsal lateral tegmental nuclei and superior olive, that abut the raphe nuclei (Fig. 1*B*), which likely explains their detection when analyzed by qPCR on dissected DR tissue. Sense probes were used as controls.

Overall, these results indicate a preferential expression of EPhA5 over other EphA receptors in the developing DR and MnR.

### EphA5 expression is dynamically regulated during raphe development

**Fig. 2.**
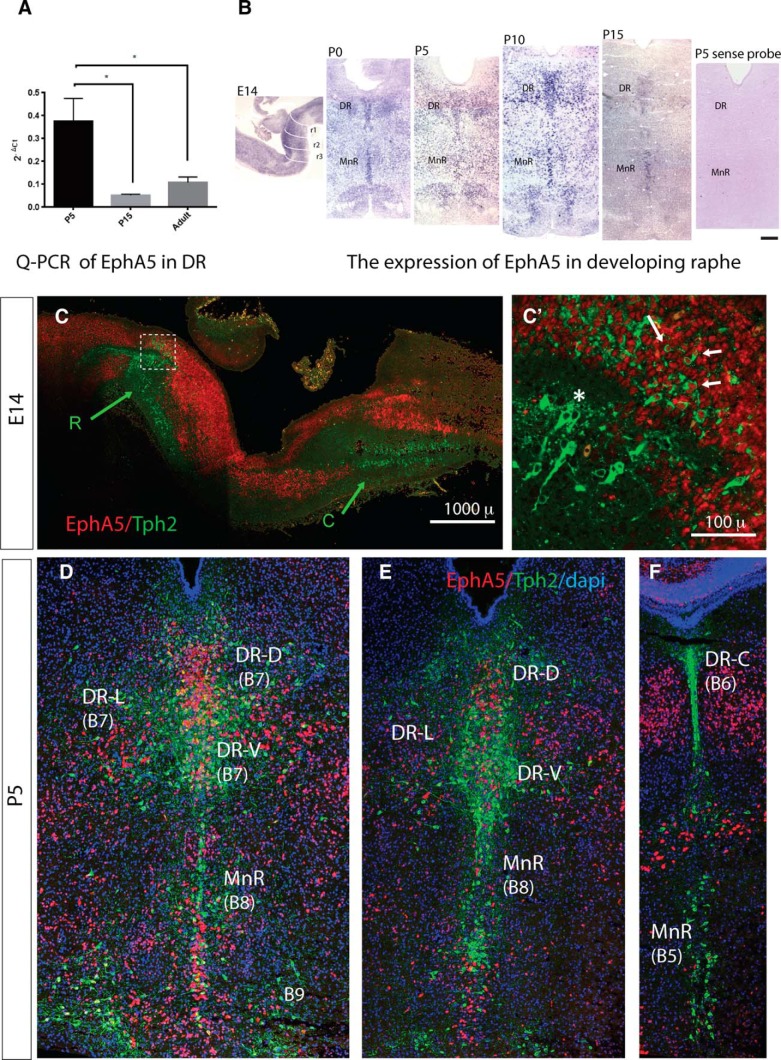
EphA5 is expressed in serotonergic neurons during embryonic and postnatal development. ***A***, qPCR of EphA5 mRNAs in DR extracts from P5 (*n* = 3), P15 (*n* = 3), and adult (*n* = 3). Relative mRNA expression was calculated as 2^–ΔCt^. Data are presented as mean ± SEM from three independent experiments, ***B***, Time course of EphA5 expression in the developing raphe was analyzed on sagittal (E14) and coronal (P0, P5, P10, P15) sections through the raphe nuclei. Note the decrease in EphA5 expression by P15. No expression is detected using the sense probe of EphA5. Scale bar = 500 μm. ***C***, Colocalization was visualized on confocal images after Tph2-immunostaining (green) and EphA5 ISH (red). Sagittal section of E14 mouse brain through the rostral (R) and caudal (C) raphe clusters that are indicated with arrows. Note that the dorsal part of the rostral cluster overlaps with EphA5 labeling, whereas the ventral part does not. ***C***′, High-power image of the boxed area in ***A***. Arrows indicate colocalized neurons (red nuclear labeling for EphA5 and green cytoplasmic labeling for Tph2); the asterisk shows Tph2^+^ neurons with no EphA5 expression. Scale bar = 1 mm (***A***), 100 μm (***A′***). D, E, F, Coronal sections of a P5 mouse hindbrain at rostral (***B***), intermediate (***C***) and caudal (***C***) levels of the raphe. Sections were counterstained with DAPI. Scale bar = 500 μm.

To evaluate the possible developmental impact of EphA5 at different stages of raphe development, we analyzed its expression timeline. Serial sagittal (E14, *n* = 4) and coronal (postnatal and adult) sections were processed for EphA5 ISH P0 (*n* > 5), P5 (*n* > 5), P10 (*n* > 5), P15 (*n* > 5), and adult (*n* = 2). EphA5 expression was detectable in the rostral raphe at E14 (Fig. 2*B*). This expression was maintained at a high level over the first week after birth and subsequently declined by P15 (Fig. 2*B*) up to adulthood, where only weak expression was detectable (not shown). To obtain a quantitative evaluation of the time course of expression, qPCR measures of EphA5 mRNA were done on DR tissue from P5, P15, and adult brains (Fig. 2*A*). This showed a significant decrease of EphA5 expression between P5 and P15 (*p* < 0.05), consistent with the ISH observation.

These results indicated that EphA5 is dynamically regulated during development, with highest expression in early postnatal life and a subsequent decline in expression by P15.

### Serotonergic raphe nuclei differ in EphA5 expression pattern

Hindbrain raphe nuclei contain a heterogeneous neuronal population that includes, in addition to 5-HT neurons, glutamatergic, GABAergic, and peptide-containing neurons. To determine whether EphA5 is specifically expressed in the serotonergic neurons, we combined fluorescent *EphA5* ISH and tryptophan hydroxylase (Tph2) immunocytochemistry (Nguyen et al. 2001; Fig. 2). At E14, 5-HT neurons have not yet achieved their full migration, making it difficult to clearly distinguish all the individual raphe cell groups other than the two main rostral and caudal clusters. However, a distinction could be made between the dorsal and ventral Tph2^+^ neurons of the rostral cluster that corresponds to the prospective DR and MnR, respectively (Fig. 2*C*). In the dorsal part, a large number of colocalized EphA5-Tph2^+^ neurons were found (Fig. 2*C′* ), whereas rare colocalization was found in the ventral part. In contrast, in the medulla, the caudal Tph2^+^ cluster appeared to be entirely segregated from the *EphA5*-expressing region.

**Fig. 3.**
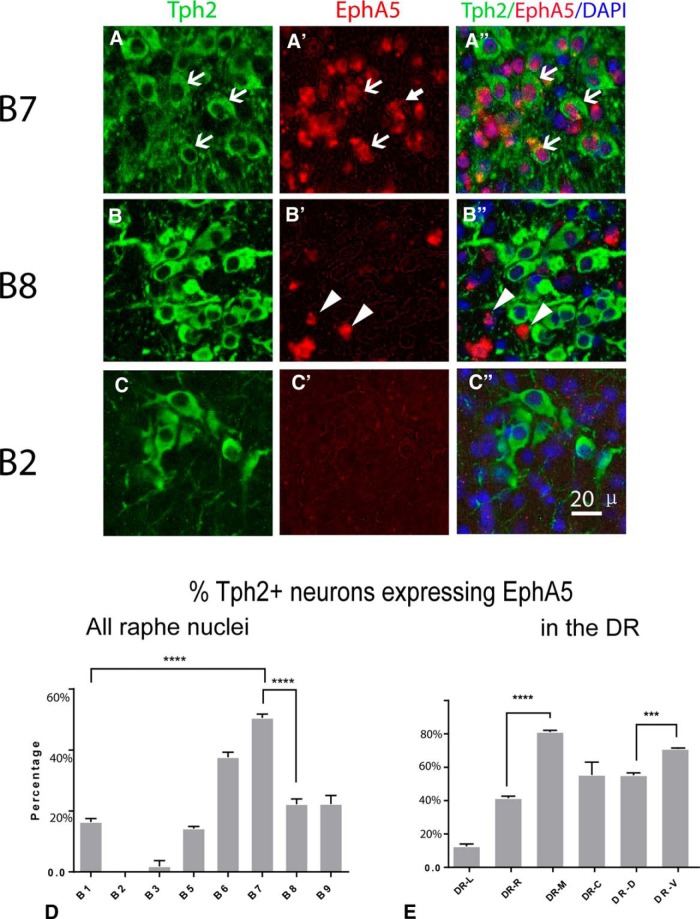
Quantification of EphA5-Tph2 colocalization in distinct raphe nucleus. ***A–C***, High-power confocal images in B7, B8, and B2, showing the difference in colocalization of EphA5 and Tph2 in different raphe nuclei. White arrows point to Tph2+EphA5 colocalized neurons; arrowheads show neuron containing only EphA5. Scale bar = 50 μm. ***D***, ***E***, Histograms summarizing the percentage colocalization among the different raphe nuclei, subdivided as the B1–B9 cell groups (***D***) and within the DR, where colocalization was compared at three rostro-caudal levels, caudal DR DR-C), middle DR (DR-M), rostral DR (DR-R), and three DR subdivisions, DR lateral (DR-L), DR dorsal (DR-D), and DR ventral (DR-V). Data are presented as mean ± SEM (*n* = 3). One-way ANOVA, ***p < 0.005 and ****p < 0.001.

A more detailed quantitative evaluation of EphA5-Tph2 colocalization was done at P5, since distinction of these components can be done clearly at this stages (Fig. 2*D*, *E*), and this postnatal developmental stage is highly relevant to the ingrowth of 5-HT axons in their targets. Colocalization of Tph2 and was evaluated quantitatively in the different subnuclei (Fig. 3; Tables 4 and 5). The highest colocalization index was found in the DR (B7, all subcomponents pooled), where more than half of the 5-HT neurons expressed EphA5 (50.2% ± 2.2%, *n* = 3, Fig. 3*A*, *D*), whereas the lowest colocalization ratio was noted in the caudal (B1–B3) raphe cell groups (Fig. 3*C*, *D*). In the MnR (B5, B8), a strong expression of *EphA5* was visible, but only a minority (14% and 22%, respectively) of the 5-HT neurons expressed *EphA5* (Fig. 3*B*, *D*). Further heterogeneous expression was observed within the DR, where colocalization was compared at three different rostro-caudal levels (Fig. 3*E*) and in three different DR subdivisions (lateral, dorsal, ventral; Fig. 3*E* and Table 5); Medially located DR neurons have a higher percentage of colocalized neurons than the laterally located DR neurons (Fig. 2*B*, *C*; Fig. 3*E*). Overall, this colocalization pattern indicates a clear topography of EphA5 expression in the raphe subgroups, with a rostral to caudal decreasing expression that was visible during embryonic stages (Fig. 2*C*) and maintained at P5 (Fig. 3). Such differences in expression of EhpA5 between the different 5-HT cell groups suggested that EphA5 signaling could be involved in the differential targeting of these various 5-HT neurons.

**Table 4.** Counts of Tph2-immunopositive and EphA5-expressing neurons and colocalized neurons in different raphe nuclei

Neuron	B1	B2	B3	B5	B6	B7	B8	B9
Tph2^+^	8.2 ± 0.9	2.8 ± 0.1	7.4 ± 0.9	5.1 ± 0.1	30.7 ± 1.9	11.2 ± 0.9	5 ± 0.4	7.3 ± 0.5
EphA5^+^	19.3 ± 3.3	3.3 ± 1.7	2.9 ± 1.5	8.9 ± 0.2	41.2 ± 2.9	23.3 ± 2.2	18.9 ± 1.6	16.9 ± 0.8
Tph2^+^/EphA5^+^	1.3 ± 0.2	0	0.1 ± 0.1	0.7 ± 0.1	11.5 ± 0.6	5.7 ± 0.4	1.1 ± 0.1	1.6 ± 0.2
Tph2 colocalized, %	16.4 ± 1	0	1.8 ± 1.9	14.3 ± 0.7	37.6 ± 1.6	50.5 ± 1.2	22.3 ± 0.2	22.3 ± 2.8

Data are mean cell numbers ± SEM/area obtained from counts done in three cases in ROI covering the different raphe nuclei (B1–B9) . All cells (validated by DAPI staining) immunolabeled for Tph2, for EphA5 and double-labeled for Tph2 and EhpA5 were counted in three different ROIs (dimension 0.225 mm^2^) for each structure and for each case and checked for colocalization. Percentage of the colabeled Tph2 neurons is indicated.

**Table 5.** Counts of Tph2-immunopositive and EphA5-expressing neurons and colocalized neurons in the dorsal raphe (B7; mean ± SEM, *n* = 3)

Neuron	DR-LW	DR-C	DR-M	DR-R	DR-D	DR-V
Tph2^+^	4.5 ± 0.6	17.8 ± 2.3	20.2 ± 1.6	21 ± 2	20.1 ± 2.1	17.9 ± 1.8
EphA5^+^	20 ± 1.2	20.6 ± 3.6	38.3 ± 4.3	18.6 ± 3.1	32.8 ± 3.5	26.2 ± 4.5
Tph2^+^/EphA5^+^	0.6 ± 0.1	7.4 ± 1.1	16.4 ± 1.4	11.3 ± 0.7	11.1 ± 1.4	12.7 ± 1.2
Tph2 colocalized, %	12.6% ± 1.5	41.5 ± 1.2	81.1 ± 1	44.4 ± 7.7	55.2 ± 1.4	70.9 ± 0.6

### Collapse response of 5-HT raphe neurons after the ephrinA application

**Fig. 4.**
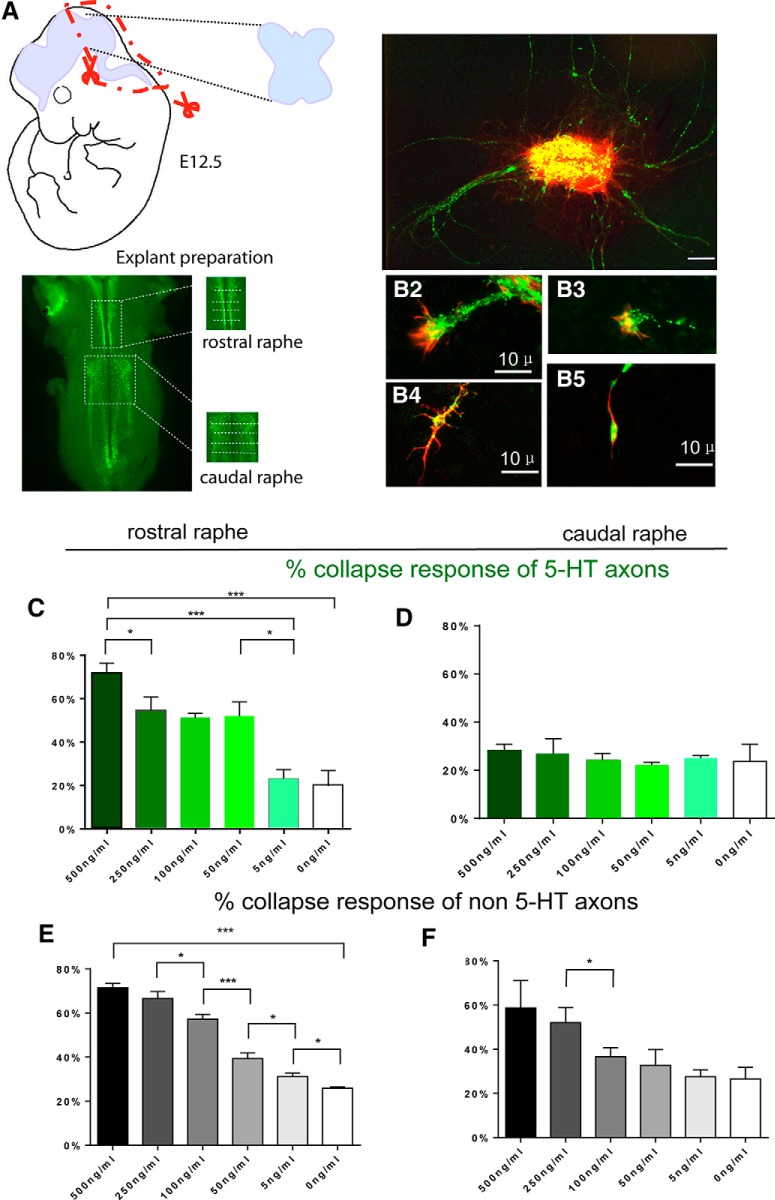
EphrinA5 induces collapse of rostral raphe serotonin axons in vitro. ***A***, Explant preparation: hindbrain was dissected as an open book from E12 embryos; the rostral and caudal raphe were dissected as depicted on a whole-mount E12 hindbrain stained for 5-HT. Scale bar = 2 mm. ***B***, Raphe explants (3DIV) were stained for 5-HT (green) and phalloidin (red). ***B1***, 5-HT^+^ axons emerging from the explant. ***B2–B5***, 5-HT–labeled growth cones displaying either a normal fan-like morphology (B2, B3) or collapsed growth cone with branch-like morphology (B4) or a long trailing process and an actin-rich retraction bulb. Scale bar = 100 μm (B1), 10 μm (B2–B5). ***C–F***, Histograms show the percentage of collapsed growth cones when explants are exposed to different concentrations of ephrinA5. ***C***, ***D***, 5-HT–labeled axons from rostral (***C***) and caudal (***D***) explants; ***E***, ***F***, non–5-HT axons from rostral (***E***) and caudal (***F***) explants (>5 explants and >100 growth cones per condition). Data are presented as mean ± SEM. One-way ANOVA, *p < 0.05, **p < 0.01, ***p < 0.005, and ****p < 0.001.

To examine the functional role of EphA5 expression on 5-HT axon outgrowth, we took advantage of the clear-cut differential expression of EphA5 between the rostral (B5–B9) and caudal-medullary (B1–B3) clusters of 5-HT neurons at embryonic ages and compared their response to application of the ligand ephrinA5. Raphe explants from E12 hindbrains were dissected as illustrated (Fig. 4*A*) and cultured for 48 h on glass coverslips. In explants from the rostral raphe, ephrinA5-FC induced the collapse of a large fraction of 5-HT growth cones (Fig. 4*B*). A dose-dependent effect was noted: 71.3% ± 4.5% of the 5-HT growth cones were collapsed at the highest concentration tested (500 ng/ml) and 45–50% at intermediate concentrations (50–250 ng/ml; Fig. 4*C*). In caudal raphe explants, ephrinA5 application did not induce a significant collapse response compared to controls at any of the concentrations tested (Fig. 4*B2*, *D* ).

Because EphA5 is also expressed in a large number of non–5-HT neurons in both the rostral and caudal raphe areas (Table 4), we also measured the collapse responses of the non–5-HT axons. The non–5-HT growth cones showed a significant dose-dependent collapse response in both rostral and caudal explants (Fig. 4*E*, *F*), thus not displaying the regional selectivity noted for 5-HT growth cones.

Overall, these experiments demonstrated that the pattern of EphA5 expression in 5-HT raphe neurons correlated with a repulsive response to the application of ephrinA5-FC. Interestingly, there was a dose–response effect, suggesting that differences in the ligand/receptor ratio could contribute to a differential targeting of 5-HT raphe neurons.

### 5-HT innervation is reduced by ectopic ephrinA expression in the amygdala

**Fig. 5.**
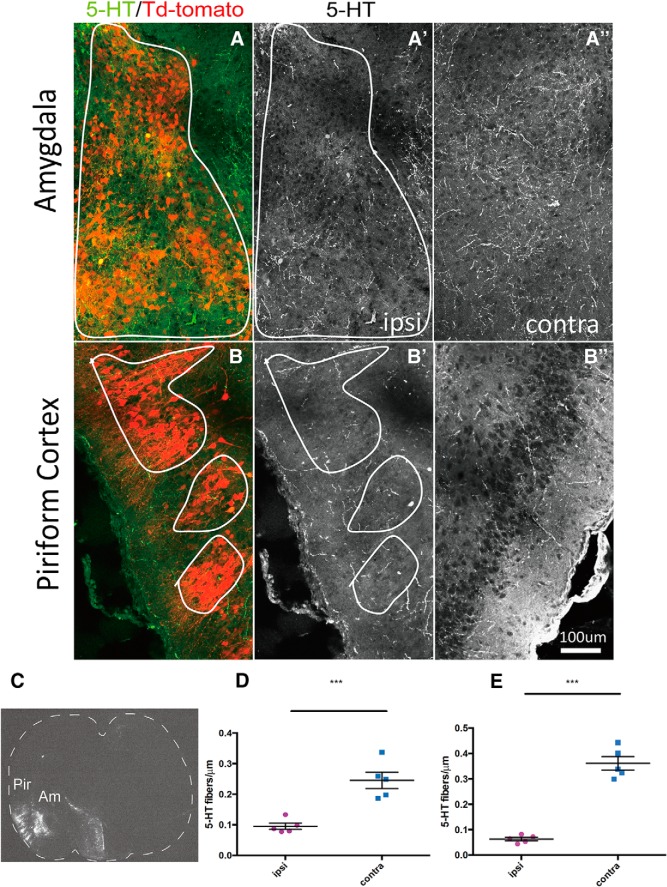
Overexpression of ephrinA3 downregulates serotonergic innervation in the amygdala and piriform cortex. ***A–B″***, Electroporated neurons were revealed by TdTomato with the ephrinA3 plasmid in the targets of Am (***A***) and Pir (***B***). Corresponding 5-HT immunocytochemistry from the electroporated (***A′***, ***B′***) and nonelectroporated (***A″***, ***B″***) sides. Note the decrease of 5-HT fibers in the outlined area compared with control side. Scale bar = 100 μm. ***C***, Target of electroporation (TdTomato) on a coronal section of P5 mouse brain at the level of amygdala. Arrows show electroporated Am and Pir. Scale bar = 1000 μm. ***D***, ***E***, Scattergram shows serotonergic fiber density in the electroporated (red) versus nonelectroporated (blue) amygdala (***D***) and piriform cortex (***E***). Overexpression of ephrinA3 resulted in a significant decrease of 5-HT fiber density compared with control groups (*n* = 5). Data are presented as mean ± SEM. Paired *t* test was used for ipsilateral vs. contralateral, ***p < 0.005.

Next, we investigated the effects of ephrinA ligands for *in vivo* 5-HT axon targeting using an overexpression strategy. We focused on the amygdala and piriform cortex, which are preferential targets of the DR 5-HT neurons (Muzerelle et al. 2016) and express only very low levels of the main EphA5 ligands, ephrinA2, ephrinA3, and ephrinA5, based on our own observations, confirming previously published reports (Deschamps et al., 2010; Gerstmann et al. 2015) and available public resources (Allen Brain Atlas, http://developingmouse.brain-map.org/gene/show/13415, 13416, 13418). This suggested that the amygdala and piriform cortex could be permissive for the ingrowth of DR axons that express high levels of EphA5 receptors. To examine this possibility, we used an *in utero* electroporation strategy to ectopically express ephrinA3 that has been shown to have the highest affinity for EphA5 (Gale et al., 1996). An ephrinA3 cDNA fragment was subcloned into a pCIG-TdTomato vector; the plasmid with or without the ephrinA3 insert was electroporated into the amygdala and piriform cortex at E12.5, and the brains were processed at P5 for 5-HT immunocytochemistry. No structural changes were observed on the electroporated/nonelectroporated side, as evaluated by Nissl staining. The electroporated cells were visible in different parts of the amygdala (basolateral and basomedial amygdaloid nucleus) and in the piriform cortex (Fig. 5*A–C*). Measures of 5-HT fiber density showed a significant decrease of 5-HT fibers on the electroporated amygdala (0.07 ± 0.01 fibers/μm) and piriform cortex (0.07 ± 0.01 fibers/μm) compared with the contralateral nonelectroporated amygdala (0.21 ± 0.03 fibers/μm) and piriform cortex (Figs. 5*D*, 7*E* ). Conversely, in the cases electroporated with the control vector, the density of 5-HT fibers was unchanged compared to the nonelectroporated side. This result indicated that ectopic expression of ephrinA3 during development can specifically reduce 5-HT raphe-amygdala innervation (*n* = 5, *p* < 0.005).

### EphrinA5 is required for the differential forebrain targeting of B7/B8 raphe neurons

**Fig. 6.**
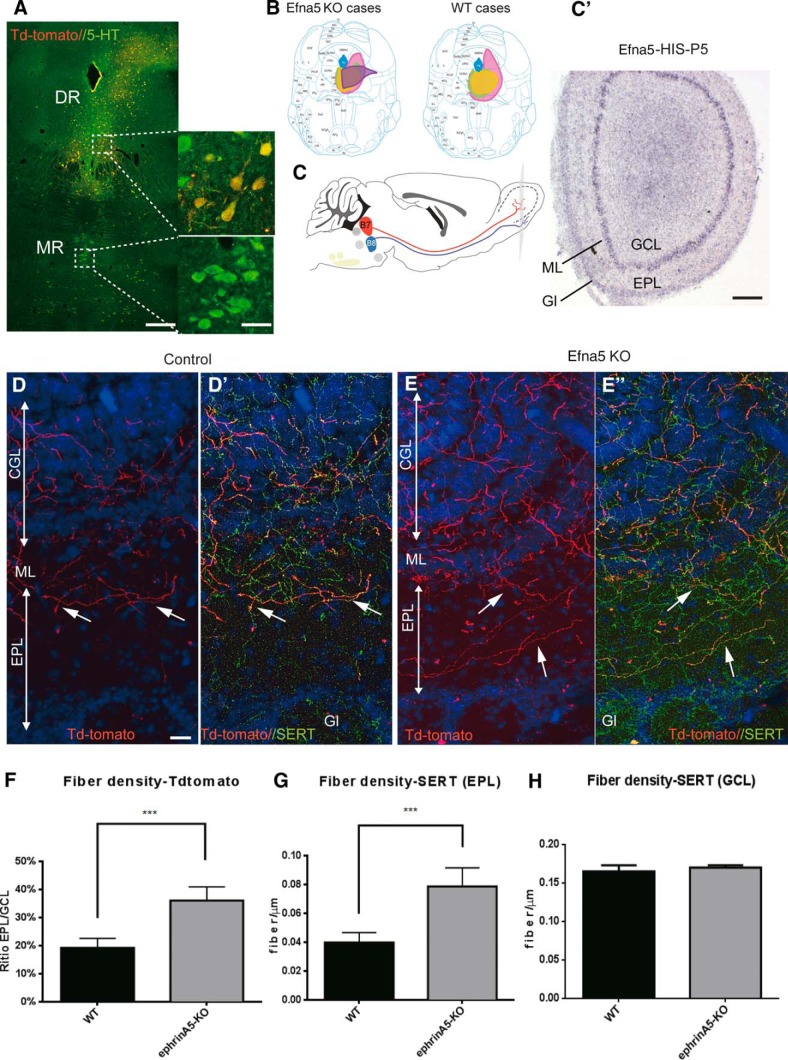
Anterograde tracing from dorsal raphe to olfactory bulb in ephrinA5^–/–^ mice. ***A***, AAV-TdTomato virus was injected in the DR. Injection sites were checked with 5-HT immunohistochemistry on the coronal raphe sections, showing 5-HT and TdTomato labeling in the DR but not in the MnR (***A***). Scale bar = 500 μm (left), 50 μm (right). ***B***, Scheme shows the extent of AAV transfection in the WT (*n* = 5) and ephrinA5 (*n* = 5) cases; the injection site was reconstructed for each case and drawn manually on representative coronal raphe sections (bregma: –4.60 mm) using a different color code for each case. ***C***, Sagittal mouse brain scheme showing the projections from the DR and MnR to the olfactory bulb (OB) targeting the inner (GCL) or outer (GL) layers, respectively. ***C′***, *In situ* hybridization of ephrinA5 (Efna5) mRNA on OB coronal section. A selective expression of ephrinA5 is observed in the mitral layer (ML). Scale bar = 250 μm. ***D–E′***, Anterogradely labeled axons (TdTomato) and SERT^+^ fibers in the OB of WT (***D***, ***D′***) and ephrinA5 KO mice (***E***, ***E′***). Most of the TdTomato were colabeled with SERT. More colabeled fibers were detected in the EPL of ephrinA5^–/–^ mice, compared with WT. Scale bar = 50 μm. ***F–H***, Histograms show the fiber densities of anterogradely labeled and SERT-labeled axons. ***F***, The density of TdTomato fibers was normalized by calculating the EPL/GCL fiber density ratio. The density of 5-HT axons was measured as linear density of SERT-labeled axons (fibers/μm) in the EPL (***G***) and CGL (***H***; ***p < 0.005).

**Fig. 7.**
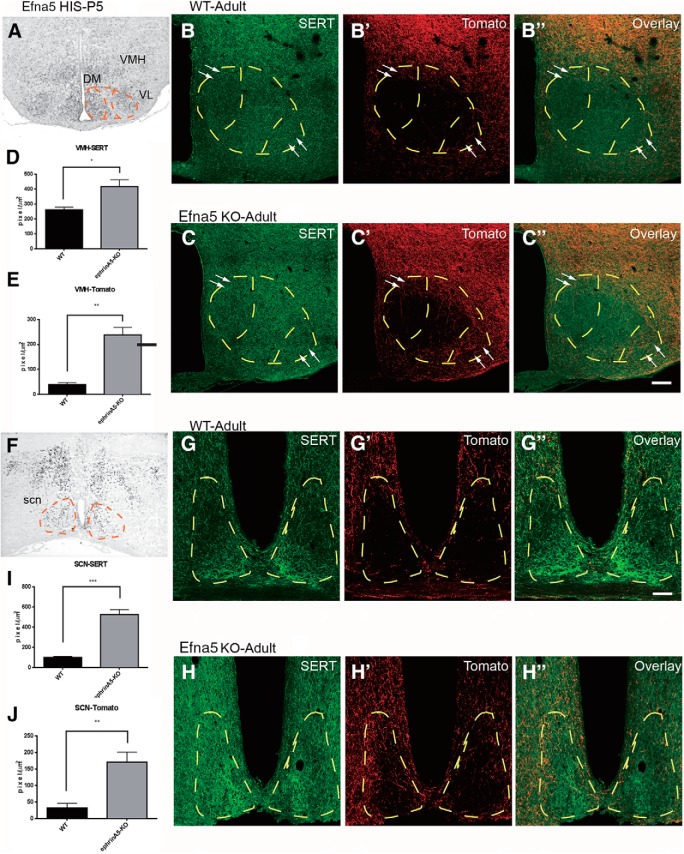
EphrinA5 expression modulates DR innervation in forebrain target. ***A***, ***F***, Expression of ephrinA5 (Efna5) in the ventromedial hypothalamus (VMH; ***A***), and the suprachiasmatic nucleus (SCN; ***F***) in coronal brain section of P5 mice. High levels of ephrinA5 were detected in the VL and DM part of VMH. Scale bar = 500 μm. ***B–B″***, ***C–C″***, ***G–G″***, ***H–H″***, Immunofluorescence images show SERT labeling and anterograde labeling of DR in adult in control (***B–B″***, ***G–G″***) and ephrinA5 KO (***C–C″***, ***H–H″***) mice. Few fibers were detected in VMH and SCN of the control cases, whereas labeled fibers were detected in larger amounts in the VMH and SCN of the ephrinA5 KO (see arrows to compare between pictures). Scale bar = 100 μm (***B–C″***), 50 μm (***G–H″***). ***D***, ***E***, ***I***, ***J***, Histograms show fiber densities anterogradely labeled and SERT^+^ axons. Fiber density was calculated as pixels per square micrometer, and data are presented as mean ± SEM. Unpaired *t* test, *p < 0.05, **p < 0.01, and ***p < 0.005.

To investigate the role of endogenous ephrins in 5-HT axon targeting, we investigated the effects of loss of function of one of the major EphA5 ligands, ephrinA5, using the ephrinA5 KO mouse model. We reasoned that 5-HT neurons with high EphA5 expression in the raphe should avoid innervating brain regions containing high levels of the ligand ephrinA5. ISH of an Efna5 riboprobe was done on serial sections of P5 brains. We focused on three main regions with high ephrinA5 expression, the olfactory bulb (OB; Fig 6*C*), the ventromedial hypothalamus (VMH), and the suprachiasmatic nucleus (SCN; Fig. 7*A*, *F*). Indeed, previous selective anterograde tracing from these DR and MnR had shown that the DR targets the granule cell layer of the OB seems arrested by the mitral cell layer, whereas the MnR targets the glomerular layer (GL) of the OB (Fig. 6*C*; Steinfeld et al. 2015; Muzerelle et al., 2016). Similarly, 5-HT DR axons consistently avoid the VMH and SCN that are instead innervated by MnR raphe neurons (Bang et al. 2012; Muzerelle et al. 2016)

Anterograde tracing of the DR neurons was done with an AAV viral vector expressing TdTomato comparing ephrinA5 KO and wild-type (WT) mice. We evaluated the extent of anterogradely labeled neurons that were 5-HT–immunopositive in the raphe (Fig. 6*A*) and the size of the injection site (Fig. 6*B*). Transfection was limited to the DR (Fig. 6*B*), and the number of transfected 5-HT cells was equivalent in the control and mutant mice. Anterogradely labeled axons were further characterized as serotonergic (or not), using SERT immunohistochemistry (Fig. 6*D′*, *E′* ).

In the OB of WT mice, anterogradely labeled axons, all of which were SERT^+^, were restricted to the granular cell layer (GCL) and appeared to be arrested at the edge of the mitral cell layer (ML) that expresses high ephrinA5 levels (Fig. 6*C*); only a few fibers entered into the external plexiform layer (EPL; Fig. 6*D*, *D′* ), confirming previous observations (Steinfeld et al. 2015; Muzerelle et al. 2016). In the ephrinA5 KO mice, DR axons did not seem to be arrested by the ML, which they crossed, arborizing into the EPL (Fig. 6*E*, *E′* ). To obtain quantitative measures normalized to the number of anterogradely labeled axons, we estimated the density of TdTomato-labeled fibers in the CGL and EPL and calculated the EPL/CGL ratio. This ratio was significantly increased in ephrinA5 KO mice compared with WT mice (Fig. 6*F*). To determine whether the overall density of 5-HT–labeled axons was modified, we measured the density of SERT-labeled axons in the CGL and EPL and found an overall increase in the density of 5-HT axons in the EPL but not in the CGL (Fig. 6*G*, *H*). This indicates that the increased EPL/CGL ratio in ephrinA5 KO mice is not the consequence of a general increase in the number of 5-HT axons reaching the OB, but rather an increase in the fraction of DR axons that cross beyond the ML.

In the same cases, we further analyzed DR anterograde labeling and global 5-HT innervation in two hypothalamic areas that show high ephrinA5 expression: the VMH (Fig. 7*A*) and the SCN (Fig. 7*F*). Both nuclei show a high level of ephrinA5 at P5 (Fig. 7*A*, *F*). In WT mice, anterograde labeling from the DR showed that neither the VMH nor the SCN was targeted by DR axons (Fig. 7*B′*, *G′* ), confirming previous observations (Bang et al. 2012; Muzerelle et al., 2016). However, these areas contained a very high density of SERT-labeled terminals that originate mainly from the MnR. In the ephrinA5 KO mice, the density of DR anterograde projection was substantially increased in both the VMH (Fig. 7*C′*, *E* ) and the SCN (Fig. 7*H′*, *J* ). Double labeling (TdTomato and SERT) showed that both 5-HT and non–5-HT DR axons contributed to this increase (Fig. 7*C″*, *H″* ). However the overall density of SERT-labeled fibers also showed a significant increase in the VMH (Fig. 7*E*, *I*).

**Fig. 8.**
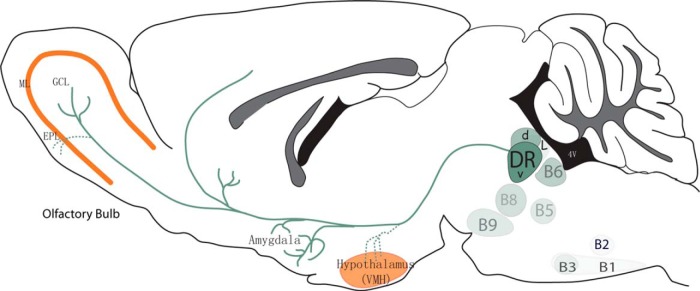
Summary diagram. This scheme shows the expression level of EphA5 in the different 5-HT raphe nuclei, indicated with different shades of green, the maximal being in the DR-V, and lowest in B1, B2. The projections of the DR 5-HT neurons to the amygdala and to the granular cell layer (GCL) of the olfactory bulb are indicated with solid lines. In the ephrinA5 knockout mice, additional projections to the ventromedial hypothalamus (VMH) and the external plexiform layer (EPL) are visible, indicated with dotted lines. High ephrinA5-expression in the mitral cell layer (ML) and VMH is indicated in orange.

These experiments indicated a requirement of ephrinA5 for the targeting of DR axons, showing that the absence of ephrinA5 results in an increased serotonergic innervation of distinctive layers of the OB and of key hypothalamic nuclei, resulting in an overall increase of the 5-HT innervation in these areas (Fig. 8).

## Discussion

Our results demonstrate for the first time a role of ephrinA signaling for the selective targeting of serotonergic raphe nuclei. We show that EphA5 is differentially expressed across the different hindbrain raphe nuclei, and that this correlates with a different repulsive action of ephrinA on 5-HT axon growth. Ectopic expression of ephrinA inhibits the ingrowth of 5-HT raphe axons in main targets of the dorsal raphe 5-HT neurons, and ephrinA5 loss of function causes a mistargeting of dorsal raphe 5-HT axons, resulting in localized increases in 5-HT innervation.

The development of raphe neurons has been well outlined by classic morphological studies in rodents (Lidov and Molliver, 1982; Wallace and Lauder, 1983); however, few insights have been obtained to date into the molecular control of axon guidance in this system. Wnt signals have been implicated in the polarity of 5-HT neurons (Fenstermaker et al., 2010), and Slit/Robo signaling influences 5-HT axon tract organization in the medial forebrain bundle (Bagri et al., 2002), but hardly anything is known about the molecular control of selective 5-HT axon targeting. This knowledge gap is likely due to a prevailing view of 5-HT neurons as a diffuse, highly collateralized system with limited specificity (Agnati et al., 2006). However, increasing evidence shows that raphe 5-HT neurons are in fact heterogeneous in their molecular identities (Wylie et al., 2010; Okaty et al., 2015; Fernandez et al., 2016), physiology (Calizo et al., 2011; Fernandez et al., 2016), genetic determinants (Jensen et al. 2008; Kiyasova et al., 2011), and connectivity (Azmitia and Segal, 1978; Commons, 2015; Muzerelle et al., 2016). In addition to the established divergence of axons arising from the rostral and caudal 5-HT neuron clusters (directed toward the forebrain and the spinal cord, respectively), there is a distinct topographic organization within the ascending forebrain projections as 5-HT axons originating from the DR and MnR occupy complementary terminal territories in the forebrain (Bobillier et al., 1976; Azmitia and Segal, 1978; Jacobs et al., 1978; Vertes et al., 1999; Muzerelle et al., 2016). Coinciding with this topographic anatomical organization, our study revealed a gradient of EphA5 gene expression, with higher EphA expression in the DR than in the MnR, and in addition a clear rostral-to-caudal and medial-to-lateral expression gradient. Thus, high EphA5 expression in DR neuronal subsets could explain why DR 5-HT neurons do not innervate brain areas that have high expression of ephrinA, and that these areas are instead innervated by 5-HT neurons from the MnR. This is particularly clear in the case of the OB, where DR and MnR 5-HT neurons are located respectively in the central (GCL) or outer (EPL, GL) layers of the OB (Steinfeld et al 2015; Muzerelle et al., 2016). The ephrinA3/5-expressing mitral cell layer might then act as a barrier to prevent DR 5-HT axons from crossing into the outer OB layers. Similarly, hypothalamic nuclei that normally receive all (suprachiasmatic) or a majority (VMH) of their 5-HT innervation from the MnR (Bang et al., 2012; Muzerelle et al., 2016) show high levels of ephrinA expression during development. The implication of ephrinA was supported by altered distribution of DR axons. In both the OB and the hypothalamus, 5-HT innervation was increased and anterogradely labeled axons from the DR were misplaced as though an inhibitory barrier was removed. Conversely, when an ephrinA ligand was ectopically expressed in a structure such as the amygdala, which is a preferential target of the DR 5-HT innervation (Muzerelle et al., 2016), the ingrowth of 5-HT raphe axons was significantly reduced. Thus, present results indicate that ephrinA5 signaling contributes to the selective targeting of 5-HT axons in the forebrain by repelling the ingrowth of 5-HT axons originating from the DR, in brain regions that are normally targeted by the MnR.

EphrinA signaling may also influence other aspects of the topography of DR; this is suggested by our observation of a difference of EphA5 expression in the medial lateral parts of the DR, which coincides with differential anatomical projections. 5-HT neurons in the lateral wings of the DR with low level of EphA expression project to regions with high ephrinA5 expression such as the lateral geniculate nucleus (Wilks et al. 2010; Muzerelle et al. 2016). It will be interesting to determine in the future how the combination of axon guidance molecules in raphe targets contributes to attracting subsets of 5-HT axons to defined brain areas/layers. In particular, we do not know what factors attract the MnR 5-HT axons to the areas that are avoided by the DR. Intriguingly 5-HT itself could contribute to the growth-promoting effects, since a defective innervation of the SCN was observed in Tph2-KO mice (Migliarini et al., 2013). The mistargeting of DR axons in the hypothalamus of ephrinA5 KO mice concerned both 5-HT and non–5-HT neurons of the DR; indeed, as noted in the present study, both cell types express EphA5. These common axon guidance cues are consistent with shared connectivity profiles: previous anatomical tracing studies showed that DR afferents frequently contain a mix of 5-HT and non–5-HT neurons (Steinbusch and Nieuwenhuys, 1981; Kiyasova et al., 2011); the latter could include glutamatergic Vglut3^+^ (Hioki et al., 2010) and GABAergic neurons (Bang et al., 2012). Thus DR neurons could share similar axon guidance mechanisms, independent of their neurotransmitter content.

EphA-ephrinA signaling is involved in several neuronal developmental processes from cell migration to synaptic maturation (Cooper et al. 2009; Cramer and Miko, 2016; Kania and Klein, 2016), although its best-known implication in neural development is for axon guidance, where both repulsive and attractive interactions have been described. Our current studies indicated a main inhibitory effect of the ephrinA ligands on 5-HT axon growth: *in vitro,* ephrinA5 induced a collapse of the growth cones, and *in vivo* ectopic expression of ephrinA3 inhibited 5-HT axon ingrowth. This corresponds to the classic repulsive forward signaling of EphA receptor activation (Kania and Klein, 2016) and is most likely due to the EphA5 receptor according to the present localization studies. However, we cannot exclude the implication of other EphAs, since transcriptional profiling of raphe neurons in embryonic and postnatal brains also reported the presence of other EphA (Wylie et al., 2010; Okaty et al., 2015), but likely expression is at levels that are too low for our ISH detection. Moreover, the loose specificity of the EphA5 receptors for ephrinA ligands and the redundancy of ephrinA expression in several brain targets (such as the mitral cells in the OB) suggest that the defects of 5-HT axon targeting observed in the ephrinA5 KO might be more pronounced in double or triple ephrinA KO mice. Such redundancy has previously been shown in the visual system (Feldheim et al., 2000).

In the visual and auditory sensory maps, Eph-ephrinA signaling acts to build a continuous topographic map (Cramer and Miko, 2016); however, present results do not indicate that this is the case in the 5-HT raphe system, where topography is much looser. Indeed, DR and MnR have different targets but do not display further topographic organization within their preferred targets. Thus, we propose that as regards the 5-HT systems, Eph-ephrinA signaling could act in a target selection process, by generating nonpermissive boundaries for the ingrowth of DR 5-HT raphe subtypes. This effect would then be more similar to that observed for the motor neurons when choosing dorsal/ventral muscle targets during development (Eberhart et al., 2004).

EphA5 expression in 5-HT raphe neurons was dynamically expressed, being maximal during axon growth in embryonic life, reaching target during the early postnatal period, and showing decreased expression, similarly to what has reported for ephrinA5 expression (Deschamps et al., 2010). Given the potential of 5-HT neurons to regenerate and grow, it will be interesting to know whether the present developmental mechanisms are reactivated after a lesion, and whether the propensity of serotonin axons to regrow (Müllner et al., 2008) is linked to their EphA content.

What could be the pathophysiological consequences of targeting defects of raphe neurons in the olfactory bulb or the hypothalamus? Our observations in ephrinA5 KO showed that mistargeting of the DR axons was correlated with a general increase of 5-HT innervation in these regions, suggesting that excitatory/inhibitory balance is compromised in these brain nuclei. Interestingly, behavioral observations conducted in ephrinA5 and EphA5 KO mice showed some phenotypes that could relate to our observations. These studies report a reduction in intermale aggression (Mamiya et al., 2008; Sheleg et al., 2015), and an increase of 5-HT levels in the hypothalamus (Mamiya et al., 2008), consistent with our observations of increased 5-HT innervation in this brain region. Interestingly, the increased 5-HT innervation that we found in the ephrinA5 KO was concentrated in the ventrolateral part (VMHVL), which is implicated in modulating aggression (Martinez et al 2008; Silva et al. 2016). Clearly, the pathophysiological consequences of SCN hyperinnervation calls for further studies on the circadian rhythms in these mutants, given the implication of 5-HT innervation to the SCN in entraining circadian rhythmicity (Versteeg et al., 2015)

Given the implication of 5-HT in a wide range of behaviors and psychiatric disorders, our study points to new gene targets that may indirectly affect 5-HT functions by changing the targeting of raphe neurons and inducing modifications of 5-HT inputs in selected brain regions.

## Synthesis

Reviewing Editor: Esther Stoeckli, University of Zurich Decisions are customarily a result of the Reviewing Editor and the peer reviewers coming together and discussing their recommendations until a consensus is reached. When revisions are invited, a fact-based synthesis statement explaining their decision and outlining what is needed to prepare a revision will be listed below. The following reviewer(s) agreed to reveal their identity: Artur Kania. Note: If this manuscript was transferred from JNeurosci and a decision was made to accept the manuscript without peer review, a brief statement to this effect will instead be what is listed below.

Unfortunately, it was not possible to send the manuscript back to both reviewers of the original manuscript submitted to J. Neurosci. However, I have carefully analyzed the response to the second reviewer's comments myself. Together, the available reviewer and myself, we feel that the revised version of the manuscript is acceptable for publication in eNeuro.
